# Quality of life of Jordanian menopausal working and retired women and its associated factors: a cross-sectional study

**DOI:** 10.12688/f1000research.125887.2

**Published:** 2024-02-06

**Authors:** Enas A. Assaf, Muntaha K. Gharaibeh, Sawsan Abuhammad, Mohannad AbuRuz

**Affiliations:** 1 Faculty of Nursing, Applied Science Private University, Amman, Jordan; 2 Maternal and Child Health Department, Faculty of Nursing, Jordan University of Science and Technology, Irbid, Jordan; 3 Faculty of Nursing, Al Ahliyya Amman University, Amman, Jordan

**Keywords:** menopausal women, QoL, Jordanian

## Abstract

**Objective:**

Life expectancy of Jordanian women has increased, indicating that the number of women entering menopause age, during the prime of their working life, will also increase. Therefore, assessments of the quality of life (QoL) of working and retired women and factors associated with overall wellbeing, are essential for the provision of quality services and care.

**Method:**

A cross-sectional study was conducted with 200 Jordanian women between the ages of 45 to 60 years old. The Utian QOL tool was used to assess the quality of life among menopausal women. Multiple regressions were used to determine predictors for QoL for the whole sample and for each group of working and retired women.

**Results:**

The study shows that the total QoL for women was 77.5 ±14.4, with a significant difference (p=.023) in total QoL and the occupational domain (p=.003) between working and retired women. Employed women with fewer chronic diseases and using frequent preventive measures had a higher QoL compared to others.

**Conclusion:**

Working itself might be an important indicator for better a quality of life among menopausal women. Better working conditions and more attention from the health care providers for the menopausal changes and the preventive measures could enhance women’s perceived QoL in addition to increasing their productivity.

## Introduction

Menopause is a normal transitional period experienced by more than 1.5 million women each year. It is characterized by cessation of the menstrual period and many other disruptive symptoms among which are joint pain, hot flashes, vaginal dryness, insomnia and general tiredness and fatigue. ^1^^,^^2^ Among other important symptoms are mood changes and depression, which is correlated with estrogen level decrease during the menopausal stage. ^3^

Menopausal age is associated with multiple morbidities and chronic diseases, particularly after the age of 60, such as hypertension, diabetes mellitus arthritis, heart diseases, breast cancer, depression, osteoporosis, chronic obstructive pulmonary diseases, and stroke. ^4^ Menopausal age is also associated with many psychological changes such as memory loss, lack of self-confidence and issues with body image. ^5^ Therefore, the menopausal stage represents a very important biological milestone in a women’s lifespan; the transition from reproductive to a non-reproductive phase is marked by cessation of menstrual period ^6^ which may have negative effects on the women’s quality of life (QoL).

The health related QoL was initially defined by WHO based on the concept of subjective perception, cultural context and value scheme. ^7^ In addition, the objective context is related to specific schemes measured for the studied population. ^8^ Women’s QoL, which could be related to several important factors such as biological as represented by hypoestrogenism, psychosocial, and cultural perspectives and might differ based on their attitudes toward menopausal changes and age which culturally could be either defined as positive or negative stage. ^9^^,^^10^

In a meta-analysis study of 14 studies among Iranian post-menopausal women, it was found that the mean QoL was higher than moderate, while the lowest QoL was reported for the physical and the sexual domain. ^11^ In one Egyptian study using a menopause specific quality of life instrument (MENQOL), it was found that physical symptoms were perceived as the most affecting to women’s QoL, and had the lowest mean score. ^12^ Another study in Iran used WHO Quality of Life-BREF (WHOQOL-BREF) and the Menopause Rating Scale (MRS) among premenopausal and postmenopausal women; QoL was found to be negatively correlated with menopausal symptoms at all WHOOL domains (physical health, psychological health and social interactions). Moreover, QoL decreased with increasing the severity of menopausal symptoms. ^13^ While in Saudi Arabia/Qatif, there were no difference in QoL domains between menopausal and postmenopausal women. ^14^ Previous studies showed that working women might have better QoL in the menopausal and postmenopausal stages than those unemployed or housewives, as they might have better access to health care, better economic stability, social interaction and empowerment which all might reduce the physical symptoms of menopause. ^9^^,^^13^^,^^15^^–^^22^ Moreover, women at this stage would be at the age of highly skilled and work role models, despite the difficulties and challenges of the menopausal symptoms. ^23^ One study measured Serbian women’s QoL using the Utian menopausal QoL scale, which has one specific domain measuring the occupational QoL and found that that employment and economic status were correlated with the occupational domain in QoL. ^17^ On the other hand menopausal symptoms shows to have adverse effect on work as what was found in UK study. ^24^

### Jordanian women at menopausal age

Bustami *et al*., ^25^ conducted a cross-sectional study to assess factors associated with onset of premature/early menopause among 409 Jordanian women. Findings revealed that the mean age of natural menopause (ANM) is 48.5±5.0, and 2.7% of the women experienced premature menopause (ANM <40) and 7.8% early menopause (ANM 40–44). Smoking was highly associated with early/premature menopause, chronic diseases or combinations of diseases were associated with average (45–52 years) or late menopause (>52 years). BMI, arthritis pain, hot flushes and inconsistent urination were significantly higher in the early and regular menopause than in premenopausal and perimenopause women.

Jaber *et al*. (2017) examined patterns and severity of menopausal symptoms among 359 women between the age of 45-65 years. The Arabic version of Menopause Rating Scale (MRS) was used. Results showed that the mean age at menopause was 49.4 years, a total of 105 women had regular menstruation (premenopausal), 49 experienced irregular cycles, and 205 had reached menopause. Women who were still menstruating regularly were found to be 1.5 times more likely to suffer from irritability, while women experiencing irregular cycles but were still menstruating, were found to be 1.63 times more likely to suffer from hot flushes, approximately 2 times more likely to suffer from physical and mental exhaustion and more likely to have sexual problems, vaginal dryness, and joint and muscular discomfort.

Severity of menopausal symptoms of Jordanian women was examined by Gharaibeh *et al*., (2010) who recruited 350 women between the ages of 45 to 55 years. Authors used the Greene Climacteric Scale to measure the severity of menopausal symptoms. The scale consists of domains of vasomotor symptoms, somatic symptoms, psychological symptoms and sexuality symptoms. Results showed that the mean age of menopause was 48.7 years. A positive association between severity of menopausal symptoms and both educational level and menopausal status; the mean score for severity of menopausal symptoms in perimenopausal women was higher than that for premenopausal women. The scores for the psychological, somatic, vasomotor and sexuality subscales were significantly higher among the perimenopausal, and postmenopausal groups compared to the premenopausal group. Perimenopausal women’s scores for all clusters were higher compared to those of postmenopausal women except for the vasomotor and its symptoms of sweating at nights, and the sexuality and its symptoms of loss of interest in sex, vaginal dryness and pain during intercourse.

An earlier study by Al-Qutob (2001) investigated types and magnitude problems associated with menopausal using exploratory study with 317 of Jordanian women between 18 and 49 years old and 185 women older than 50 years. The mean and median age of onset of menopause were 47.5 and 49 years. Chronic diseases were found to be present among menopausal women such as urinary tract infection, hypertension, diabetes, iron deficiency anemia, reproductive tract prolapse.

In Jordan life expectancy at birth has increased for females to 78.6 in 2020. ^26^ However the retirement age for women in Jordan is 55 for women where women can ask for early retirement at early age between 50 and 52 years old and can still benefit from the governmental pension. ^27^

It is clear from the review of the available studies that there is a gap in studies from Jordan that addressed the quality of life of menopausal working women. Therefore, the study comes to fill the gap by assessing the quality of life of Jordanian working and retired women and its associated factors. The importance of the study comes from the fact that the life expectancy at birth of Jordanian women has increased from 61.98 years in 1971 to 76.45 years in 2019, ^28^ indicating that the numbers of women in perimenopause, transitional phase and postmenopausal ages will increase and we are expected to see more working, active women involved in the society and enjoy a positive QoL. Findings of this study will help women live a productive life, and health care professionals who can provide supportive services that enhance working women’s QoL.

## Methods

### Ethics

The study was approved by the Institutional Review Board (IRB) of Applied Science Private University, Jordan. The informed participant consent was obtained along with the data collection with maintaining participant privacy, confidentiality, and anonymity which was built into the design and the process of the instrument. The cover letter included with the questionnaire stated the study purpose and that the completion and submitting of the questionnaire was indication of consenting to the study.

A cross-sectional study was conducted with 200 Jordanian women during the period from 5 ^th^ January to 5 ^th^ March 2021. Sample size was determined using power analysis software for the following parameters: alpha 0.05, power of 95%, moderate effect size 0.3. The needed sample size was 196 participants; 200 questionnaires were completed and submitted. The main inclusion criteria were working and retired women between the age of 45 to 60 years old. The women were excluded if they suffered from any mental disorders since these will impact the QoL. The women were recruited from main authors or research assistants who were employed from the main authors. These women were recruited based on information provided on leaflets, which were distributed in health centers and hospitals. The leaflets included information on the researchers and how to contact them.

Women were recruited using social media and emails **.** This research utilized Google Forms for data collection; a link was sent by the authors to participants via social media, such as Facebook, Instagram. Google forms is an application in the form of a template or worksheet that can be used independently or together for the purpose of obtaining information. Google form is very easy to understand and use and is available in the Arabic language. The form took 15 minutes to fill out.

### Measurement tool

The questionnaire consisted of three parts; the demographic part which consisted of seven questions on age in years, employment status (employed, retired), social status, level of education, chronic diseases, menopausal stage (pre, menopausal and postmenopausal), and finally number of children. All variables were categorical except for age and number of children, which were continuous. The second part was the menopausal information and preventive measures, which consisted of the perceived menopausal health related information, (very good, moderate, know some, not good), sources of menopausal information (social media, family/friend, TV, health personnel) and health promotion and disease prevention measures (BSE, monogram, pap smear, routine checks). The third part was the QoL measures, the study used the Utian QoL developed by Utian *et al*. (2018). The tool consists of 23 items divided into four main domains: occupation domain (7 items), health domain (7 items), emotional domain (6 items) and sexual domain (3 items). The original tool was validated with a diverse sample of peri- and postmenopausal women using the Short Form-36 ^29^ (well established inventory of QoL). For the QoL domains, confirmatory factor analyses were conducted with a second sample of 270 women. The original scale is in the English language and showed to be valid and reliable. Cronbach’s alpha for the 23-item scale as a whole was 0.830, occupational domain was 0.83, health domain 0.73, emotional domain 0.64 and for the sexual domain 0.79. ^29^

For our study, the scale was translated to the Arabic language using the World Health Organization standard (translation and back translation process). ^30^ This method consisted of the following steps: forward translation; expert back-translation; pretesting and cognitive interviewing and producing the final version. A panel of three experts in psychiatry, mental health nursing, nursing education and women’s health from Jordan University of Science and Technology and Applied Science University. These experts were chosen based on their experience and were tasked with checking the content validity and the process of translation and back translation. The Arabic version was then pilot tested with 25 menopausal women, which were not included in the sample. These women were recruited by research assistants and have the same inclusion and exclusion criteria of the participants of this study and were not entered into the main data of the project. Results of the pilot testing revealed that it is simple to read, and completion of the tool required approximately 10 minutes. The Cronbach reliability coefficients for the translated version for the total scale was 0.85, it was 0.80 for the occupational domain, 0.69 for the emotional domain, 0.62 for the health domain, and 0.50 for the sexual domain.

### Data analysis

Data was analyzed using the SPSS software for Windows version 25 (IBM Corp, USA, RRID:SCR_016479). Descriptive statistics including means, standard deviation, numbers, and frequencies were used to describe the sample characteristics. Means and standard deviation were used to describe each domain and the total QoL. The t-test was used to show the differences in the four domains for working and retired women. Frequencies and percentage were used to describe the response for each item of QoL. Chi-square test was conducted to show the difference in each item for QoL for working and retired women. Multiple regression was conducted to determine predictors for QoL for the whole sample and for each group of working and retired women.

## Results

### Demographic and health background characteristics

Table 1 presents the demographic characteristics of the sample as the mean age of women was 50.5±4.8 years old. Employed women were 117 (58.5) and retired women were 83 (41.5).

**Table 1.** Sociodemographic characteristics of the sample (N=200).

Characteristics	N (%)
**Employment status**	
Employed	117 (58.5)
Retired	83 (41.5)
**Age (**M±SD)	
Employed	(48.1±3.0)
Retired	(53.7±5.0)
**Working as**	
Administrator	40 (20.0)
Academic	49 (24.5)
Health profession	55 (57.5)
Others	58 (28.5)
**Social status**	
Currently Married	174 (87.0)
Others	26 (11.5)
**Level of education**	
Bachelor and more	117 (58.5)
Less than Bachelor	83 (41.5)
**Perceived family income as**	
Satisfactory	39 (19.5)
Somehow satisfactory	75 (37.5)
Not satisfactory	86 (43.0)
**Chronic disease**	
Hypertension	58 (29.0)
Thyroid gland dysfunction	20 (10.0)
Severs back pain	51 (26.5)
Diabetes mellitus	33 (16.5)
Cardiac diseases	8 (4.0)
Varicose veins	29 (14.5)
Bowel irritability	10 (5.0)
Breast cancer/other cancer	4 (2.0)
None	71 (35.3)
**Menopausal stage**	
premenopausal	68 (34.9)
Menopausal	52 (26.0)
post-menopausal	80 (40.0)
**Number of children**	
None	12 (6.0)
One	6 (3.0)
Two	19 (9.5)
Three	32 (16.0)
Four	57 (37.0)
Five and more	74 (37.0)

M: mean, SD: standard deviation.

### Information and preventive measures

Women reported acceptable information level regarding the menopausal stages and changes 134 (67%). The majority receive their information from social media, rather than their health care providers, 61 (30%). For preventive measures, self-examination breast checks that were done regularly the previous year, was 63 (31.5) and those who had a Mammogram examination once during the previous year was 40 (30.0). As shown in Table 2.

**Table 2.** Health promotion and information of the sample (N=200).

	N (%)
**Perceived Menopausal health related information**	
Very good	62 (31.0)
Moderate	72 (36.0)
Know some information	38 (19.0)
Not good need more information	28 (14)
**Sources of menopausal information**	
Social media	61 (30.0)
Family/friends	54 (27.0)
TV and audio media	46 (23.0)
Health staff	39 (19.5)
**Health promotion and disease prevention measures**	
Self-breast examination regularly last year	63 (31.5)
Mammogram examination once last year	40 (30.0)
Pap smear test once last year (prevention)	38 (19.0)
Regular gynecology checkup every 6 month	31 (15.5)

### Domains of QoL

The total QoL for women was 77.5±14.4, which is higher than the average total universal quality of life (UQoL). ^19^ There is a significant difference (p=.023) in total QoL between working (80.5±12.7) and retired women (73.4±15.7). The highest domain mean score was for the occupational domain 25.9±6.4, followed by the emotional domain 22.3 ±4.8, and health domain 21.5±5.0, while the lowest mean score was among the sexual domain 7.6±2.6. There was a significant difference in occupational health (p=.003) between working (27.2±5.3) and retired women (24.1±7.4), see Table 3.

**Table 3.** t-test for differences in Quality-of-Life domains between working and retired women.

Domain	Whole sample	Working (M±SD)	Retired (M±SD)	P value
Occupational domain	25.9± 6.4	27.2±5.3	24.1±7.4	.003
Health domain	21.5±5.0	22±5.1	20.9±4.9	.562
Emotional domain	22.3 ±4.8	22.9±4.3	21.3±5.3	.082
Sexual domain	7.6 ±2.6	8.2±2.3	6.9±2.7	.063
Total QoL	77.5±14.4	80.5±12.7	73.4±15.7	.023

t-test, p≤.05 based on no-equal variance.

### Description of QoL among menopausal women

Descriptive statistics was used to describe the women's response to the QoL tool. The highest response was for **“**I am currently experiencing physical discomfort or pain during sexual activity with my partner” 141 (71%), “I think my work benefits society” 129 (65%), and “I'm not happy with my appearance” 124 (62%). However, the lowest score response was reported for “I do exercise at least 3 times a week” 15 (8%), “I feel like I'm not satisfied with my sex life” 21 (11%), and “I feel in good shape” 38 (19%).

There was a significant difference in QoL between working and retired women in seven questions. Items were, “I am able to control the important things in my life” (p=.024), “I think my work benefits society” (p=.007), “I am satisfied with my romantic life” (p=.007), “I get anxious frequently” (p=.003), “I am proud of my professional achievements” (p=.006), “I keep setting personal goals for myself” (p=.006), and “I keep setting professional goals” (p=.001) ( Table 4).

**Table 4.** Description of QoL for Whole Sample for Menopausal women **(N=200)**.

Items	Not agree		Neutral		Agree	
1. I can control things that are important to me	27	14%	80	40.0%	93	47%
2. I feel challenged to prove myself and my work	37	19%	75	37.5%	88	44%
3. I believe my work benefits society	20	10%	51	25.5%	129	65%
4. I feel like I'm not content with my sexual life	131	66%	48	24.0%	21	11%
5. I am content with my romantic life	78	39%	68	34.0%	54	27%
6. I have gotten a lot of personal appreciation and recognition in my community or at my job	31	16%	69	34.5%	100	50%
7. I'm unhappy with my appearance	34	17%	42	21.0%	124	62%
8. I think my diet is not nutritionally sound	49	25%	89	44.5%	62	31%
9. I feel able to control my eating behaviors	43	22%	101	50.5%	56	28%
10. I do exercise at least 3 times a week	134	67%	51	25.5%	15	8%
11. My mood is generally depressed	29	15%	72	36.0%	99	50%
12. I frequently experience anxiety	39	20%	77	38.5%	84	42%
13. Most things that happen to me are out of my control	35	18%	65	32.5%	100	50%
14. I am content with the frequency of my sexual interaction with my partner.	67	34%	70	35.0%	63	32%
15. I am currently experiencing physical discomfort or pain during sexual activity with my partner	20	10%	39	19.5%	141	71%
16. I believe I have no control over my physical health	32	16%	83	41.5%	85	43%
17. I am proud of my occupational achievements	23	12%	58	29.0%	119	60%
18. I consider my life stimulating	24	12%	79	39.5%	97	49%
19. I continue set a new personal goals for myself	26	13%	69	34.5%	105	53%
20. I expect that good things will happen in my life	23	12%	74	37.0%	103	52%
21. I feel physically well	26	13%	111	55.5%	63	32%
22. I feel physically fil	69	35%	93	46.5%	38	19%
23. I continue to set new professional goals	39	20%	76	38.0%	85	43%

### Predictors of QoL for whole sample of menopausal women

Multiple regressions test was conducted to determine the predictors of QoL among menopausal women. The model was significant (F= 3.5, p<. 001). This means that many factors predicted QoL among women; these were employment status/currently employed (B=.175, p=.046, r ^2^=.34), number of chronic diseases (B=-.198, p=.004, r ^2^=-.260), and using preventive measures (B=.177, p=.011, r ^2^=.222). Employed women with fewer chronic diseases and using frequent preventive measures had higher QoL compared to others. The three variables explained 54% in the variance of QoL. See Table 5.

**Table 5.** Predictors of QoL for the whole sample for menopausal women.

		Unstandardized Coefficients		Standardized Coefficients	t	Sig.
		B	Std. Error	Beta		
1	(Constant)	86.645	14.269		6.072	0.000
	How old are you	-0.070	0.304	-0.024	-0.231	0.818
	What is your employment status now?	-5.124	2.552	-0.175	-2.007	0.046
	What is the nature of your business	-0.439	0.749	-0.047	-0.587	0.558
	number of chronic diseases	-5.990	2.043	-0.198	-2.932	0.004
	What is your marital status?	-0.069	1.521	-0.003	-0.046	0.964
	What is your education level?	1.298	1.088	0.092	1.194	0.234
	Do you think your monthly income is sufficient for the requirements of your life/the lives of those you care about?	1.020	1.315	0.052	0.776	0.439
	How do you assess your situation now at any stage?	0.937	1.591	0.056	0.589	0.557
	the number of your children	0.215	0.916	0.018	0.235	0.814
	The number of your loads	0.426	1.261	0.026	0.338	0.736
	What are your sources of information about the age of safety (you can choose more than one answer?	0.434	0.922	0.031	0.471	0.638
	Using preventive measures	5.122	2.006	0.177	2.553	0.011
	Did you use hormonal contraceptives during your reproductive life?	1.084	1.945	0.038	0.557	0.578

^a^ Dependent variable: Total QoL.

### Predictors of QoL for working and retired menopausal women

Multiple regression tests were conducted to determine the predictors of QoL among working and retired menopausal women. These models were significant for working women (F=4.45, p=.001) and retired women (F=6.52, p<.001). The only significant predictors for QoL for working women were number of chronic diseases (B=-.206, p=.006, r ^2^=-.267) and using of preventive measures (B=.196, p=.003, r ^2^=.194) and income (B=0.204, p=0.037, r ^2^=0.134). This means working women with less chronic diseases and using preventive measures had higher QoL compared to retired women. The only significant predictors for QoL for retired woman were education (B=.199, p=.04, r ^2^=.231), menopausal status (B=.199, p=.04, r ^2^=.222), and using of preventive measures (B=.217, p=.045, r ^2^=.194). This means retired women with higher education, who reached postmenopausal, and using preventive measures had higher QoL compared with other women. See Table 6.

**Table 6.** Predictors of QoL for working and retired menopausal women.

		Unstandardized coefficients		Standardized coefficients	t	Sig.
		B	Std. Error	Beta		
Working	(Constant)	31.342	11.026		2.842	0.005
	How old are you	-0.048	0.224	-0.028	-0.216	0.829
	Education	-1.475	1.222	-0.132	-1.206	0.230
	number of Chronic diseases	-6.778	2.415	-0.264	-2.806	0.006
	Income	1.444	0.685	0.204	2.109	0.037
	Menopausal status	0.771	0.813	0.111	0.948	0.345
	Number of children	-0.290	0.478	-0.066	-0.607	0.545
	Number of pregnancies	-0.141	0.659	-0.023	-0.214	0.831
	Menopause information	-0.226	0.571	-0.040	-0.396	0.693
	Source of information	1.638	1.059	0.151	1.547	0.125
	preventive measures	5.086	2.441	0.196	2.084	0.040
Retired	(Constant)	27.760	11.331		2.450	0.017
	How old are you	-0.154	0.218	-0.104	-0.708	0.481
	Education	11.904	3.749	0.352	3.176	0.002
	number of Chronic diseases	-2.706	1.847	-0.158	-1.465	0.147
	Income	0.075	1.132	0.007	0.067	0.947
	Menopausal status	5.852	2.818	0.298	2.077	0.041
	Number of children	1.311	0.879	0.191	1.493	0.140
	Number of pregnancies	-0.668	1.090	-0.081	-0.612	0.542
	Menopause information	-1.181	0.811	-0.175	-1.455	0.150
	Source of information	1.034	0.719	0.150	1.437	0.155
	preventive measures	6.834	3.351	0.217	2.039	0.045

## Discussion

Our study showed that the total mean score for quality of life of working and retired women was more than the UQoL average (77.5±14.4). The highest score was reported for the occupational domain, followed by the emotional domain, with the lowest being among the sexual domains.

There were significant differences between working and retired women in each of the occupational domains and the total QoL scores. These differences could be to domain questions that showed a significant difference between the two groups of women related to their work benefits to society (p=.007), being proud of their professional achievements (p=.006), setting personal goals for themselves (p=.006), and in setting professional goals (p=.001). This suggests that working serves as a good source of self-satisfaction, planning future goals, sense of achievements and recognition. This is consistent with that found in China study, ^30^ which assessed the QoL between retired and working people, to find out that working has a positive correlation with both social interactions, family interactions and leisure time. As retired women may lack the sense of achievements and may feel that their professional goals and careers have ended. Furthermore, retired women usually face changes in socioeconomic status and lifestyle. Moreover, they might lose the daily socialization with people, work interactions, and challenges of their work environment, which all could enhance the feelings of well-being. This reflects the findings of other study in USA, on the effect of retirement on ones’ QoL. ^31^

Our study showed no significant differences between working and retired women in the emotional domain. This might be related to the fact that the average age of the study sample of retired women is 53 years old, by which at this age Jordanian women have many responsibilities towards their family needs, through the role of grandmother as well as caring for their elderly parents and in-laws. These responsibilities might give them a great satisfactory feeling and emotional stability that can minimize the bad feelings of retirement, in addition to filling their time with many other responsibilities than work. ^32^ In addition, women going through menopause are likely preoccupied with the menopausal symptoms such as hot flashes which might be a priority issue. ^33^

The predictors for total QoL among menopausal women in this study were currently working with few chronic diseases, good income, and using frequent preventive measures; these factors explained better QoL compared to others (p=0.046, 0.004, 0.011 respectively). One study in Australia that reviewed the relationship between menopausal age and chronic disease and comorbidity, found a strong negative relationship between QoL and chronic diseases. ^34^ It is expected that chronic diseases in general had a negative effect on Qol looking at the overall complaints, limited physical activity and the overall psychological effect. ^35^ In one early study conducted in Jordan, the majority of menopausal women complained of chronic diseases, while only 29% reported that they are feeling “good”. ^33^ Working women appear to be more satisfied socially and have a more meaningful life, and responsibilities. As well as their time being filled with achieving their career plans and objectives, ^36^ reported that Jordanian working women have better leisure time since they have better income. Preventive measures like self-check breast examination, pap smear tests, mammograms among and regular gynecological check ups could be all widely affected by women’s level of education, income and availability of services, which was found in different cultures and studies. ^37^^–^^40^ Working women could have a better opportunity to conduct any of the preventive measures than others; the availability of health insurance, better access to health care services and higher awareness of the importance of conducting preventive measures. ^41^ Menopausal QoL was not directly connected previously with using preventive measures, rather it related to health-related issues, problems and disease prevention and how those could affect the overall health of aging women, which was discussed by Pertynska-Marczewska & Pertyński. ^42^

Income satisfaction reflects women’s access to health services and follow up on health preventive measures as well as a better sense of high self-esteem and empowerment, which was discussed previously with several previous studies. ^43^^–^^45^ Women’s employment would increase the overall family income and empowerment, one study among Jordanian women found that women with higher family income reported less severe menopausal symptoms compared to those with low family income. ^36^

Our study shows that retired women with higher education, who reached postmenopausal, and used preventive measures had a higher QoL compared to other women. Higher educated women have better opportunities and more understanding of the symptoms of menopausal changes, which makes the adaptation a lot easier and more acceptable. ^33^^,^^46^^–^^48^ A study conducted in Iran found positive correlations between levels of education and occupational status, with better scores of psychological symptoms, they found that women might have a better understanding of menopausal symptoms and better awareness related to the psychological and physiological changes of the menopause. ^49^ Moreover, women in postmenopausal stage reported less severe symptoms compared with pre and peri-menopausal stage. Studies among Jordanian women found that the severity of the menopausal symptoms is positively associated with the women’s level of education, and the menopausal stage. ^32^^,^^36^ On the other hand, Jordanian women have positive attitudes towards menopause in that they would have more time to enjoy their Ramadan fasting without any interruptions and pray regularly. Moreover, Jordanian women appreciate that transitional period since it gives them a status of wise women, which would help them to participate in decision making and empower them. ^32^ A higher level of education positively affects women’s health promotion and health prevention behaviors. ^38^

Our study shows that only 19.5% of women received their information about menopausal changes from health care professionals, even though women in this period require more information about the changes in the physiological and psychological status in order to better cope with them. ^49^

## Conclusion

In summary, working menopausal women have a significantly better QoL than retired women among both total QoL and among the occupational domains. Major predictors for working women for better QoL were employment, fewer chronic diseases and frequent use of preventive measures. While significant predictors for better QoL among retired women were higher level of education, being in the postmenopausal stage, and using preventive measures.

### Implications

Health care providers can pay more attention towards the menopausal changes, chronic disease health promotion and disease prevention for working women, to increase their work productivity and achievements. Policy makers should pay more attention towards improving the working conditions that support the transitional period for menopause, to increase women’s productivity and improve their health status during these important life stages.

Increasing the Jordanian women life expectancy would suggest adding more working years for women, as retired women seem to perceive a lower QoL than working women, therefore this study’s results might be used as a valid indicator for revisiting the retirement age law.

It’s recommended for further research on women’s perception of the best retirement age and the influence of their menopausal stage on their work productivity.

### Limitations

This study has limitations that rise from the convenience nonprobability sample that limited the generalizability of the findings. The fact that the study used Google Form for data collection has eliminated women with poor reading comprehension and those who are not familiar with the use of technology or do not have access to computers or mobiles. Findings are also limited to the use of a translated scale developed for women from western cultures, which may not have captured the cultural aspects of the QoL of Jordanian women.

## Data availability

### Underlying data

Figshare: Data Set Menopause Women.xlsx. https://doi.org/10.6084/m9.figshare.20822032.v2. ^50^

This project contains the following underlying data:

• data set menopause.xlsx
• menapause study questionnaire.docx

Data are available under the terms of the Creative Commons Attribution 4.0 International license (CC-BY 4.0).
