# Supplementary material for: Quality of life of Jordanian menopausal working and retired women and its associated factors: a cross-sectional study
Source: F1000Res. 2024 Feb 6;11:1189. Originally published 2022 Oct 18. [Version 2] doi: 10.12688/f1000research.125887.2 (PMC11130584; doi:10.12688/f1000research.125887.2)
Supplement: Peer review history

# Reviewer report: Reviewer response for version 2

**DOI:** 10.5256/f1000research.162087.r309814

**Published:** 2024-08-01

**Authors:** Harmeet Kaur Kang

**Affiliations:** 1 Chitkara University, Punjab, India

I have gone through the revised manuscript. The manuscript has been revised as per my comments in the previous review report. The revised version of the  paper is approved.

Is the work clearly and accurately presented and does it cite the current literature?

Partly

If applicable, is the statistical analysis and its interpretation appropriate?

Partly

Are all the source data underlying the results available to ensure full reproducibility?

Partly

Is the study design appropriate and is the work technically sound?

Yes

Are the conclusions drawn adequately supported by the results?

Partly

Are sufficient details of methods and analysis provided to allow replication by others?

Partly

Reviewer Expertise:

Women Health, Cardiovascular disease prevention

I confirm that I have read this submission and believe that I have an appropriate level of expertise to confirm that it is of an acceptable scientific standard.

# Reviewer report: Reviewer response for version 2

**DOI:** 10.5256/f1000research.162087.r244304

**Published:** 2024-03-14

**Authors:** Camille Cronin

**Affiliations:** 1 University of Essex, Colchester, England, UK

I am happy with the amendments.

Is the work clearly and accurately presented and does it cite the current literature?

Partly

If applicable, is the statistical analysis and its interpretation appropriate?

I cannot comment. A qualified statistician is required.

Are all the source data underlying the results available to ensure full reproducibility?

No

Is the study design appropriate and is the work technically sound?

Partly

Are the conclusions drawn adequately supported by the results?

No

Are sufficient details of methods and analysis provided to allow replication by others?

Partly

Reviewer Expertise:

Women's health

I confirm that I have read this submission and believe that I have an appropriate level of expertise to confirm that it is of an acceptable scientific standard.

# Reviewer report: Reviewer response for version 1

**DOI:** 10.5256/f1000research.138240.r223920

**Published:** 2024-05-27

**Authors:** Zalikha Al-Marzouqi

**Affiliations:** 1 Oman College of Health Sciences, North Batinah Branch, Oman

Title: I think the title is good and reflecting of the study.

 Abstract: Good, just in 'Method' add Cronbach's alpha that you did for your tool in arabic version. Also in 'Results', authors can add some brief information about the findings related to factors associated with overall wellbeing.

 Keywords: Good. 

 Introduction: the authors wrote a clear introduction and tough recent studies in literature review. The authors also identified the knowledge gap and the significance of the study. 

 Methods: the selected design is matching the aim and objective of the study and it is appropriate. Inclusion and exclusion criteria identified, which is good. The tool used was explained in detail. However, the authors need to slightly revise the language and grammar to make it more readable. 

**Dependent variable,** the authors should make sure that they mention dependent variables of this study.

**Independent variables,** the authors should make sure that they mention independent variables of this study.

 Tool and its validity: Clear and good. 

 Data analysis: good and clear, just to add 2 sentences about data management / control. 

 Results: regarding the descriptive statistics, it is good and clear. However, I am not a statistician to understand other type of statistics. Thus I can not provide comments.

 Discussion: good and clear, it covered different angles of the results. 

 Conclusion: no comments, it supports the results of this study.

Is the work clearly and accurately presented and does it cite the current literature?

Yes

If applicable, is the statistical analysis and its interpretation appropriate?

I cannot comment. A qualified statistician is required.

Are all the source data underlying the results available to ensure full reproducibility?

Yes

Is the study design appropriate and is the work technically sound?

Yes

Are the conclusions drawn adequately supported by the results?

Yes

Are sufficient details of methods and analysis provided to allow replication by others?

Yes

Reviewer Expertise:

Maternal Health

I confirm that I have read this submission and believe that I have an appropriate level of expertise to confirm that it is of an acceptable scientific standard.

# Reviewer report: Reviewer response for version 1

**DOI:** 10.5256/f1000research.138240.r216514

**Published:** 2023-11-21

**Authors:** Harmeet Kaur Kang

**Affiliations:** 1 Chitkara University, Punjab, India

**General Comments:** The topic of the paper is deemed important and relevant, focusing on Menopausal Quality of Life (QOL). However, there are notable concerns regarding grammar, sentence formation, and the overall association of Menopausal QOL with menopausal symptoms. The paper lacks an exploration of this critical relationship, which is a significant gap in the study.

**Specific Comments:**

 Menopausal QOL and Symptoms: The primary outcome variable, Menopausal Quality of Life, is highlighted, but the authors have not adequately addressed the association between Menopausal QOL and menopausal symptoms. This gap diminishes the overall impact of the study, and it is recommended that the authors explore and discuss this relationship in more detail.

**Tools and Content Validity:** The initial sections of the tools were reportedly self-prepared by the authors; however, there is a lack of information regarding the content validity of these tools. It is essential to provide evidence of the validity of the tools used in the study to ensure the reliability of the results.

**Statistical Tests:** A thorough review by a statistician is recommended, particularly for certain tables that appear to lack coherence. The application of statistical tests should be carefully examined to ensure accuracy and relevance. Any discrepancies or inconsistencies in the tables need to be addressed and corrected.

**Strengthening the Discussion Section:** The discussion section could be strengthened by providing a more comprehensive analysis of the global perspective on menopausal quality of life. The authors have mainly focused on the Jordanian perspective, neglecting a broader view that could enhance the generalizability and applicability of the study findings.

**Grammar and Sentence Formation**: The manuscript requires careful proofreading for grammar and sentence formation. Ensuring clarity in expression and adherence to grammatical rules will significantly enhance the readability of the paper.

 In conclusion, addressing these concerns will contribute to the overall improvement of the paper. Emphasizing the association between Menopausal QOL and menopausal symptoms, validating the tools used, reviewing statistical tests, broadening the global perspective, and enhancing the clarity of language will collectively elevate the quality of the research article.

Is the work clearly and accurately presented and does it cite the current literature?

Partly

If applicable, is the statistical analysis and its interpretation appropriate?

Partly

Are all the source data underlying the results available to ensure full reproducibility?

Partly

Is the study design appropriate and is the work technically sound?

Yes

Are the conclusions drawn adequately supported by the results?

Partly

Are sufficient details of methods and analysis provided to allow replication by others?

Partly

Reviewer Expertise:

Women Health, Cardiovascular disease prevention

I confirm that I have read this submission and believe that I have an appropriate level of expertise to confirm that it is of an acceptable scientific standard, however I have significant reservations, as outlined above.

# Response

**Published:** 2024-01-30

**Authors:** Enas Assaf

**Affiliations:** Applied Science Private University, Jordan

Comments from the reviewer

 Response

**General Comments**: The topic of the paper is deemed important and relevant, focusing on Menopausal Quality of Life (QOL). However, there are notable concerns regarding grammar, sentence formation, and the overall association of Menopausal QOL with menopausal symptoms. The paper lacks an exploration of this critical relationship, which is a significant gap in the study

***Thank you very much for your review and your comments. Regarding the menopausal symptoms and its association, this was part of the study but since the main goal was studying the difference between retired and working menopausal women, symptoms show no significance. Therefore, we did not elaborate on that and did not focus on the symptoms rather than the overall quality domains, which would in itself, reflect the symptoms in each of the quality of life studied domain.***

***English editing was considered and checked. Thank you .***

**Tools and Content Validity**: The initial sections of the tools were reportedly self-prepared by the authors; however, there is a lack of information regarding the content validity of these tools. It is essential to provide evidence of the validity of the tools used in the study to ensure the reliability of the results.

***Thank you , content validity was checked in the tool and highlighted in the paper with yellow***

**Statistical Tests**: A thorough review by a statistician is recommended, particularly for certain tables that appear to lack coherence. The application of statistical tests should be carefully examined to ensure accuracy and relevance. Any discrepancies or inconsistencies in the tables need to be addressed and corrected

***All the statistics were carried out by Expert in statistics***

**Strengthening the Discussion Section**: The discussion section could be strengthened by providing a more comprehensive analysis of the global perspective on menopausal quality of life. The authors have mainly focused on the Jordanian perspective, neglecting a broader view that could enhance the generalizability and applicability of the study findings.

***Thank you for your comments, in the discussion each point were analyzed based on both Jordanian and supported by global perspectives taking several international studies from different culture, among which some were highlighted in the text.***

# Reviewer report: Reviewer response for version 1

**DOI:** 10.5256/f1000research.138240.r191964

**Published:** 2023-09-07

**Authors:** Camille Cronin

**Affiliations:** 1 University of Essex, Colchester, England, UK

A cross-sectional study of 200 Jordanian women aged 45-60 years using a QOL tool to assess quality of life in menopausal women. 

 A range of literature is used and some of which is dated, some may correspond to the tools used but other literature should have been more up to date and more international. Menopause is a global issue.

 The study design look appropriate but I imagine that with the world count the author has had to make a decision to cut certain sections out and this I feel has affected the presentation of tool, and what stats have been chosen to present. Perhaps the author(s) need to be more specific in what they what tp present with corresponding literature to support. 

 I am not a statistician and unable to comments on the other points. 

 I do feel this is a contemporary issue to be reporting and this paper is looking at working women and those moving to retirement. I feel again this is not reflective globally as the age of working women is  growing and working past the age of 60. In Jordan this may be different but this needs to be contextualised - what is the average retirement age and so on.

 I think the discussion session is short and does not correspond with the amount tot data - perhaps the author has been limited by the word count. 

 Limitations have been reported which is also good.

 I do feel the paper is limited in the literature which I mentioned above.

Is the work clearly and accurately presented and does it cite the current literature?

Partly

If applicable, is the statistical analysis and its interpretation appropriate?

I cannot comment. A qualified statistician is required.

Are all the source data underlying the results available to ensure full reproducibility?

No

Is the study design appropriate and is the work technically sound?

Partly

Are the conclusions drawn adequately supported by the results?

No

Are sufficient details of methods and analysis provided to allow replication by others?

Partly

Reviewer Expertise:

Women's health

I confirm that I have read this submission and believe that I have an appropriate level of expertise to confirm that it is of an acceptable scientific standard, however I have significant reservations, as outlined above.

# Response

**Published:** 2024-01-30

**Authors:** Enas Assaf

**Affiliations:** Applied Science Private University, Jordan

Comments

 response

 A range of literature is used and some of which is dated, some may correspond to the tools used but other literature should have been more up to date and more international. Menopause is a global issue.

***Thank you for your comments, the following literature is added in the introduction :***

***The introduction section has been revised and updated literature was added as follows:***

***This was added in the introduction “Menopausal age is also associated with many psychological changes such as memory loss, lack of self-confidence and issues with body image” and the reference added***

1. ***Camille Cronin, Catherine Hungerford & Rhonda Lynne Wilson (2021) Using Digital Health Technologies to Manage the Psychosocial Symptoms of Menopause in the Workplace: A Narrative Literature Review, Issues in Mental Health Nursing, 42:6, 541-548, DOI: 10.1080/01612840.2020.1827101 . (ref. no…..)***

***This was added in the introduction***

***“Moreover, women at this stage would be at the age of highly skilled and work role model, despite the difficulties and challenges of the menopausal symptoms” reference: ***

1. ***House of Commons Women and Equalities Committee, Menopause and the workplace, First Report of Session 2022–23. Available at chrome-extension://efaidnbmnnnibpcajpcglclefindmkaj/https://www.elaweb.org.uk/sites/default/files/docs/HC%2091%20-%20Menopause%20and%20the%20workplace%20-%20EMBARGO.pdf. acceded on September 12, 2023.***

***Another recent systemic review study ref was cited as no ( ) related to menopausal working women and QoL.***

1. ***S. Theis, S. J. Baumgartner, H. Janka, A. Kolokythas, C. Skala & P. Stute (2023) Quality of life in menopausal women in the workplace – a systematic review, Climacteric, 26:2, 80-87, DOI: 10.1080/13697137.2022.2158729.***

***The following stidies were added in the introduction numbered as (….. )***

1. ***Krishnapriya, P. B., et al. "Assess the quality of life among postmenopausal women." International Journal of Advances in Nursing Management (2023).***
2. *** Studies in Sauid Arabia ( ref no)  ***

***Aljarudi, S. , Al-Jumah, H. and Al-Jumah, A. (2023) Evaluating the around and after Menopausal Symptoms and Personal Perception of Quality of Life among Women in Qatif, Saudi Arabia, Attending the Hospital and PHCs. Open Journal of Obstetrics and Gynecology, 13, 280-290. doi: 10.4236/ojog.2023.132029.***

  

1. *** We added the following sentence highlighted in red as “On the other hand menopausal symptoms shows to have adverse effect on work as what was found in UK study” line***

***Ref***

***Faubion, S. S., Enders, F., Hedges, M. S., Chaudhry, R., Kling, J. M., Shufelt, C. L., ... & Kapoor, E. (2023, June). Impact of menopause symptoms on women in the workplace. In Mayo Clinic Proceedings (Vol. 98, No. 6, pp. 833-845). Elsevier.***

 The study design look appropriate but I imagine that with the world count the author has had to make a decision to cut certain sections out and this I feel has affected the presentation of tool, and what stats have been chosen to present. Perhaps the author(s) need to be more specific in what they what tp present with corresponding literature to support

***Really appreciated, the cutting will impact the accuracy of the paper***

 I am not a statistician and unable to comments on the other points. 

***Well respect***

 I do feel this is a contemporary issue to be reporting and this paper is looking at working women and those moving to retirement. I feel again this is not reflective globally as the age of working women is  growing and working past the age of 60. In Jordan this may be different but this needs to be contextualised - what is the average retirement age and so on.

***Thank you for the note, this comment was well considered by adding the following in the introduction under the Jordan section***

***In Jordan life expectancy at birth had increased for females to 78.6 in 2020  insert here( worldometer, 2021). However the retirement age  for women in Jordan is  55 for women where women can ask for early retirement at early age between 50 and 52 years old and can still benefit from the governmental pension  insert ref here ( BAKER, 2018).***

***Ref: to be added in the article***

***BAKER, N. (2018). What are the retirement ages around the world? https://www.sbs.com.au/news/what-are-the-retirement-ages-around-the-world#:~:text=Retirement%20ages%2C%20or%20the%20age%20when%20some%20sort,years%20for%20men%20and%2063.7%20years%20for%20women.***

***worldometer. (2021). world lifeexpectancy at birth https://www.worldometers.info/demographics/life-expectancy/#countries-ranked-by-life-expectancy***

 I think the discussion session is short and does not correspond with the amount tot data - perhaps the author has been limited by the word count. 

***Thank you for the note, we actually considered discussing the main points and results looking to the considered limitation***

 Limitations have been reported which is also good.

***Thank you***

 I do feel the paper is limited in the literature which I mentioned above.

***Thank you considered and updated introduction section***

 Are all the source data underlying the results available to ensure full reproducibility?

***Data sources are available in the reference section highlighted***
